# n-3 PUFA Improve Emotion and Cognition during Menopause: A Systematic Review

**DOI:** 10.3390/nu14091982

**Published:** 2022-05-09

**Authors:** Davide Decandia, Eugenia Landolfo, Stefano Sacchetti, Francesca Gelfo, Laura Petrosini, Debora Cutuli

**Affiliations:** 1 IRCCS Fondazione Santa Lucia, Via Ardeatina 306, 00179 Rome, Italy; eugenia.landolfo@uniroma1.it (E.L.); stefano.sacchetti@uniroma1.it (S.S.); f.gelfo@hsantalucia.it (F.G.); laura.petrosini@uniroma1.it (L.P.); debora.cutuli@uniroma1.it (D.C.); 2 Department of Psychology, Sapienza University of Rome, Via dei Marsi 78, 00185 Rome, Italy; 3 Department of Human Sciences, Guglielmo Marconi University, Via Plinio 44, 00193 Rome, Italy

**Keywords:** menopause, menopausal transition, ovariectomy, n-3 PUFA, anxiety, depression, cognition

## Abstract

Women show an increased risk of cognitive impairment and emotional disorders, such as anxiety and depression, when approaching menopause. Data on risk and protection factors have yielded robust evidence on the effects of lifestyle factors, such as diet, in preserving emotional and cognitive functioning. This review focused on the effects of omega-3 polyunsaturated fatty acids (n-3 PUFA) on anxiety, depression, and cognition during the menopausal transition. This systematic review considered all articles published until 31 December 2021, and the search was performed on two databases, PubMed and Scopus. The fields of interest were “menopause”, “n-3 PUFA” and “emotional and cognitive aspects”. Out of the 361 articles found on PubMed and 283 on Scopus, 17 met inclusion criteria. They encompassed 11 human and 6 animal studies. Most studies reported relieved depressive symptoms in relation to n-3 PUFA intake. While controversial results were found on anxiety and cognition in humans, n-3 PUFA consistently reduced anxiety symptoms and improved cognition in animal studies. Taken together, n-3 PUFA intake shows beneficial effects on emotional and cognitive behaviours during menopause transition. However, further investigations could increase knowledge about the effectiveness of n-3 PUFA on psychological well-being in this delicate period of feminine life.

## 1. Introduction

### 1.1. Menopause as a Delicate Phase of Women Life

Throughout the various stages of life, from menarche to menopause, women experience dramatic fluctuations in the levels of sex hormones such as estrogen and progesterone [1]. Estrogens influence neuronal functions in a variety of ways, from neurotrophic and neuroprotective actions to alteration of neurotransmission [2,3]. Estrogen receptors (ERs) are widely distributed in the brain, especially in the areas that control memory and executive functions [4,5].

In a report on menopause of the World Health Organization (WHO), some guidelines on terms to be used were provided (1981) [6]. In particular, the WHO Scientific Group recommended the following definitions:

- Menopause: “the permanent cessation of menstruation resulting from loss of ovarian follicular activity”;
- Perimenopause (or climacteric or menopausal transition): “the period immediately prior to the menopause (when the endocrinological, biological and clinical features of approaching menopause commence) and at least the first year after the menopause”;
- Postmenopause: the period “from the menopause, although it cannot be determined until after a period of 12 months of spontaneous amenorrhea has been observed”.

In addition to these definitions, the WHO Scientific Group highlighted that “the term premenopause is widely used in an ambiguous manner, either to refer to the one to two years immediately before the menopause or to refer to the whole reproductive period prior to the menopause” (1981) [6].

Despite the WHO guidelines, different and partial definitions of menopausal transition are used in scientific papers, although all of the labels have in common the presence of vasomotor, cognitive and mood manifestations (see Box 1).

Box 1 **The most common symptoms of menopause.**

BOX 1. During the climacteric, middle-aged women can often suffer from menopause-related symptoms. Among these, the primary ones are vasomotor symptoms (as hot flushes and night sweats), somatic complaints, weight gain, slowed metabolism, chills, sleep disturbances, mood disorders, anxiety, memory problems, cognitive decline, “brain fog”, thinning hair, dry skin, vaginal dryness, sexual dysfunction, and urinary disorders, all changes negatively affecting women’s quality of life. In the months or years leading up to menopause, menstrual cycle length and frequency become irregular, and follicle stimulating hormone concentration rises in response to decreased concentrations of estrogens. As the menopausal transition progresses, menstrual cycles and ovulation are missed and ultimately stop.

This period of change represents for women a period of great vulnerability given by endogenous factors, such as hormonal changes, and exogenous factors, such as changes in social status.

During the menopausal transition period, firstly, the ovarian follicular function reduces and hypothalamic-pituitary-ovarian axis function deteriorates [7]. Subsequently, the levels of estrogens, as estradiol (E2), and progesterone first become fluctuating, and then decrease, whereas levels of follicle stimulating hormone increase [8,9]. These hormonal fluctuations have a significant impact on female body and may be responsible for behavioral, cognitive, and mood changes [10]. In particular, estrogens play a crucial role in the synthesis and metabolism of the neurotransmitters involved in depression, such as serotonin, dopamine and norepinephrine [11]. Namely, serotonin synthesis and availability are increased by E2 [12]. Thus, menopausal women have imbalances in homeostatic regulation of different biological systems, with alterations in hypothalamic-pituitary-adrenal (HPA) axis, renin-angiotensin-aldosterone system, and serotoninergic pathways. Disruption to these systems, in combination with the hormonal fluctuations is retained to promote a state of neuroinflammation that contributes to the development of depression [13,14].

The menopausal transition is usually accompanied by the presence of some cognitive deficits, and women usually complain memory problems, such as forgetfulness and “brain fog” [15]. The literature suggests that lowered estrogen levels may be a factor in the possible connection between menopause and dementia. In fact, lack of estrogens over the long term enhances oxidative stress, increasing brain aging and leading to cognitive impairment [16]. In fact, ERs are widely present in the areas implicated in memory and cognitive functions. Namely, ER-β is expressed mostly in the cerebral cortex and hippocampus, and ER-α is mostly expressed in cholinergic neurons of the basal forebrain [17]. E2 seems to elicit a trophic factor in the hippocampus and basal forebrain [18] and mediate interactions among neurotransmitters in the frontal cortex [19], all activities relevant for memory and executive functioning. Estrogens increase even the neurogenesis in the dentate gyrus of hippocampus [20].

Natural menopause is detected after 12 months of amenorrhoea not associated with any pathological cause [21]. However, the menopause can also be induced by radiation, chemotherapy, illnesses and surgery.

The menopausal transition most often begins between ages 45 and 55 and usually lasts about seven years, even if can be as long as 14 years. The average age of spontaneous menopause is 51.5 years in the world and 51.2 in Italy [22]. Population studies suggest that menopause onset depends on many features, such as age of menarche, ethnicity, socioeconomic status, body mass index, family history and lifestyle factors (i.e., smoking, dietary quality, physical activity and oral contraceptive use) [21,23,24].

This period is experienced by women not only as a biological process but also as an “age of life” characterized by important changes. Although it is a completely normal physiological phenomenon, and not at all a pathology, for women the menopause is a new and difficult condition to live with, since the physiological symptoms of menopause bring in women emotions of embarrassment and shame, a sense of personal inadequacy, insecurity and low self-esteem influencing so the social and family role of each woman [25]. Taken together, the physiological and psychological symptoms of menopause have a relevant impact on quality of life of women. In postmenopausal women, quality of life seems to be significantly diminished, given menopausal symptoms experienced daily (hot flushes, vaginal dryness, urinary complaints, uterine bleeding, emotional dysregulation and cognitive deficits) affect mostly work and social activities, quality of partnership, mood, concentration, and sleep quality [26].

Similarly to women, female rodents also exhibit some form of reproductive senescence (estropause), characterized by gonadal hormonal fluctuations, dysregulation of the hypothalamic-pituitary-gonadal axis, morpho-functional changes in the ovaries, and irregular fertility [27]. However, only 30–40% rodents develop an anestrous state, characterized by low levels of ovarian steroids, and a hormonal profile similar to that of menopausal transition in women [28]. Rodents tend to maintain mature ovarian follicles in reproductive senescence, unlike the menopausal women in which the ovarian failure is complete [27]. For these reasons, to reproduce human menopause, bilaterally ovariectomized (OVX) rodents are the animal model of choice [29].

Understanding the effects of reproductive aging on anxiety, depression, and cognition in middle age and beyond is a topic of great interest given that there is an increased risk of developing dementia once menopause is reached [16,30].

To treat the cognitive and emotional consequences of menopause some therapies have been advanced. Hormone replacement therapy (HRT), also known as hormone therapy (HT), is considered the primary treatment to counteract menopausal symptoms. However, given the potential health risks (breast cancer, cardiovascular diseases) associated with HRT use [31,32,33], many women choose complementary and alternative medicine (CAM) for symptoms management. CAM interventions for menopause can be divided into main two groups [34]:

- Mind-body practices: hypnosis, cognitive-behavioral therapy, relaxation, biofeedback, meditation, and aromatherapy, mindfulness;
- The use of natural products: herbs, vitamins, minerals, and dietary supplements.

Among most used dietary supplements there are omega-3 polyunsaturated fatty acids (n-3 PUFA).

### 1.2. n-3 PUFA Dietary Intake

Fatty acids are components of lipids and cell membranes in the form of phospholipids playing multiple roles in the body. They are also important energy substrates which encompass about 30% of total energy intake and are stored in adipose tissue (when in excessive amounts).

**Figure 1:**
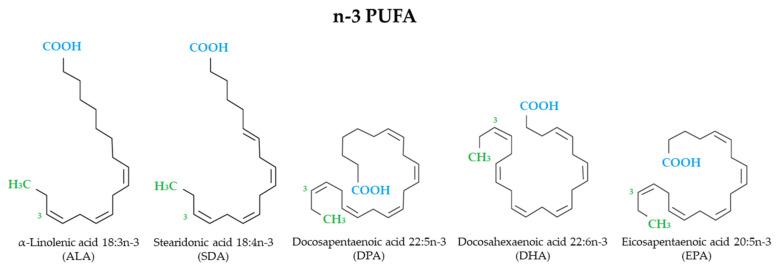
Structural formulae of n-3 PUFA.

Fatty acids are made up of chains of carbon atoms, with a methyl group (CH3) at one end [the omega (ω) or n-end] and a carboxyl group (COOH) at the other end. The carbon chains of fatty acids contain no (saturated fatty acids), one (monounsaturated fatty acids) or more (polyunsaturated fatty acids, PUFA) double bonds. There are different types of PUFA: n-6 PUFA and n-3 PUFA according to the position of the first double bond relative to the end portion of the molecule. n-3 PUFA, which comprise alpha-linolenic acid (ALA), stearidonic acid (SDA), docosapentaenoic acid (DPA), docosahexaenoic acid (DHA), and eicosapentaenoic acid (EPA) (Figure 1), are essential dietary nutrients and key modulators of many neural functions throughout life [35,36,37,38,39]. Among their multiple mechanisms of action n-3 PUFA are involved in membrane composition changes which in turn affect membrane order, formation of lipid rafts, intracellular signaling processes, gene expression, and production of both lipid and peptide mediators. n-3 PUFA inhibit many aspects of inflammation (as leucocyte chemotaxis, adhesion molecule expression, leucocyte-endothelial interactions, production of prostaglandins and pro-inflammatory cytokines, and activation of the pro-inflammatory transcription factor nuclear factor κB). In addition, EPA and DHA give rise to anti-inflammatory and inflammation resolving mediators, as resolvins [40,41].

Under typical western dietary conditions, human cells have a poor content of n-3 PUFA. ALA is the largest dietary source of n-3 PUFA [42] but, since it cannot be endogenously synthesized by humans, it must be obtained from plant sources, such as flaxseeds, cranberry seeds, walnuts, almonds, nuts, kiwi seeds, canola oil, soybeans, and chia seeds [43]. Other n-3 PUFA can be synthesized from ALA [44] but, luckily, their intake may occur from other dietary sources, such as meats, salmon, fish oil, and some types of algae and crustaceans [45].

As previously reported, cell membranes contain a high proportion of n-3 PUFA and control many neurobiological processes, such as neurotransmission, neuroplasticity, and inflammation. Increased n-3 PUFA intake has beneficial effects on brain health and function throughout the lifespan [46,47,48]. Many longitudinal and cross-sectional studies on healthy individuals showed that increased intake of n-3 PUFA has anxiolytic [49] and antidepressant [50] effects, and is associated with improved cognitive performance [51,52] and lower risk of dementia [48,53]. n-3 PUFA could exert these effects in many different ways, such as increase of brain serotonin levels [54,55], increase of hippocampal brain-derived neurotrophic factor (BDNF) levels [54], enhancement of neurogenesis in the hippocampal dentate gyrus [56,57], mitigation of activation of the HPA axis in response to stressors [58,59], and reduction of neuroinflammation [60,61].

Nevertheless, n-3 PUFA efficacy on menopausal symptoms is unclear. This systematic review was aimed at investigating the relationship between n-3 PUFA and anxiety, depression, and cognition during menopause in animal models and humans.

## 2. Materials and Methods

### 2.1. Protocol

**Figure 2:**
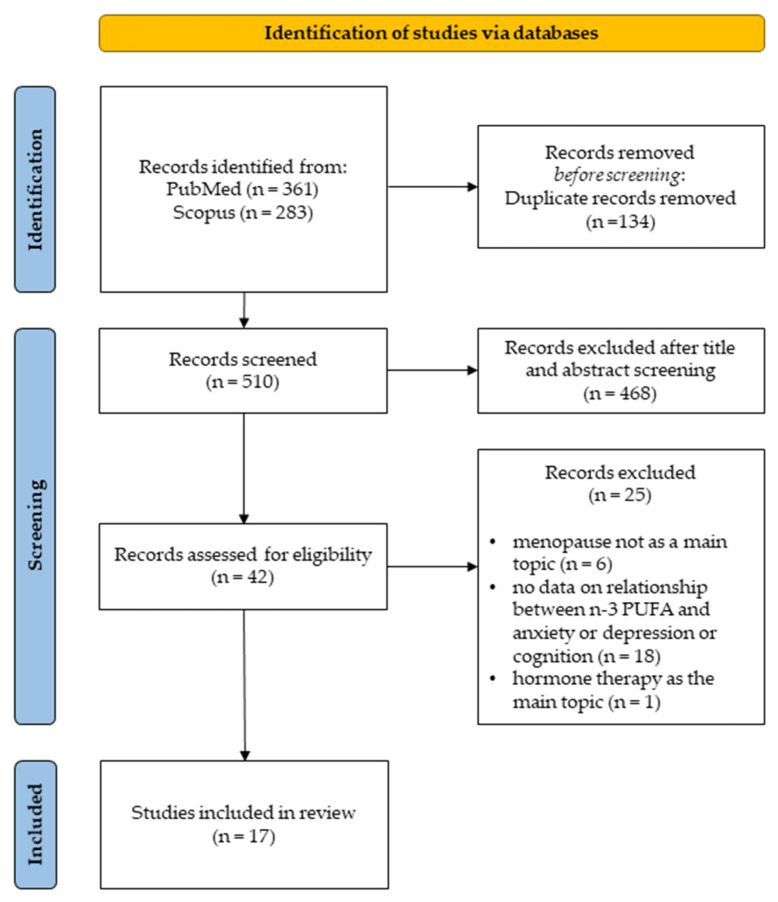
Search flow diagram based on PRIMA guidelines.

The present systematic review is consistent with the Preferred Reporting Items for Systematic Reviews and Meta-Analyses (PRISMA) guidelines [62]. It is not registered on a public database. The PRISMA flowchart of the study selection is summarized in Figure 2.

### 2.2. Search Strategy and Study Selection

A systematic literature search was performed on PubMed and Scopus to identify articles published from 1 January 1960 to 31 December 2021, focused on the following fields of interest: “menopause”, “n-3 PUFA” and “cognitive and affective aspects”. Boolean operators were used in order to create a focused search strategy. After keywords selection, the search was performed within “All Fields” in both PubMed and Scopus.

PubMed advanced search method: ((Ovariectomized OR Perimenopaus* OR Premenopaus* OR Postmenopaus* OR Menopaus*) AND (Fatty Acid OR Omega-3 OR Fish Oil OR DHA OR EPA OR ALA) AND (Behav* OR Cogniti* OR Anxiety OR Depress* OR Memor*)) NOT (Review OR Meta-analysis).

Scopus advanced search method: ((Ovariectomized OR Perimenopaus* OR Premenopaus* OR Postmenopaus* OR Menopaus*) AND (Fatty Acid OR Omega-3 OR Fish Oil OR DHA OR EPA OR ALA) AND (Behav* OR Cogniti* OR Anxiety OR Depress* OR Memor*)) AND NOT (Review OR Meta-analysis).

The screening was performed independently by three different authors.

### 2.3. Inclusion and Exclusion Criteria

The PICOS model was used to determine the inclusion criteria [63]:

- P (population): “women in menopausal transition and ovariectomized rodents”;
- I (intervention): “n-3 PUFA dietary intake and n-3 PUFA supplementation”;
- C (comparators): “control group and placebo”;
- O (outcome): “emotional and cognitive outcomes”;
- S (study design): “observational studies, clinical and preclinical trials”.

In order to stick to the research field, articles in which the primary focus targeted on cardiovascular symptoms, obesity, breast cancer, tumours and osteoporosis, and studies that did not explore the effects of n-3 PUFA were excluded. Further, we excluded articles analyzing menopausal systemic symptoms without considering at least one of the following dependent variables (Figure 2):

- Anxiety;
- Depression;
- Cognition.

### 2.4. Data Extraction

After determining inclusion and exclusion criteria and selecting the articles to include in this systematic review, data were extracted. Regarding rodent studies [64,65,66,67,68,69] the following data were extracted: study design, age, strain, sample size, ovariectomy details, n-3 PUFA treatment details, behavioral and biochemical analyses, behavioral and biochemical results (Table 1 and Table 2). Regarding human studies [70,71,72,73,74,75,76,77,78,79,80] the following data were extracted: study design, menopause definitions, sample size, age, ethnicity, exclusion criteria, n-3 PUFA treatment details, behavioral and biochemical analyses, and main results (Table 3, Table 4 and Table 5).

## 3. Results

### 3.1. Selected Studies

**Figure 3:**
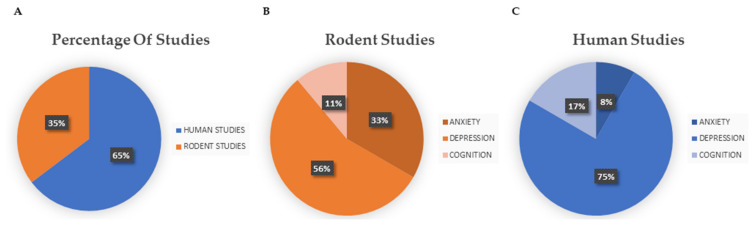
(**A**) Percentages of human and rodent studies included in this systematic review (six rodent and eleven human studies); (**B**) percentage of rodent studies divided by main topic (three studies on anxiety and depression, two studies on depression alone, and one study on cognition); (**C**) percentage of human studies divided by main topic (one study on anxiety and depression, eight studies on depression alone, and two studies on cognition).

PubMed research produced a total of 361 articles, while Scopus search produced a total of 283 results. After excluding 134 duplicate records, 510 papers were screened. After selection based on reading the abstracts, 42 publications were selected. Out of these, 25 publications were discarded after reading the full text since they did not match our inclusion criteria, and the remaining 17 publications (Figure 3A), encompassing 6 rodent (Figure 3B) and 11 human studies (Figure 3C), were included in the present systematic review. In 4 of these 17 articles, both anxiety and depression parameters were measured. In 10 articles, only depression parameters were evaluated, and in the remaining 3 articles cognitive functions were evaluated.

### 3.2. Effects of n-3 PUFA on Anxiety

Regarding anxiety, the literature research produced three articles on rodents and one on humans.

Preclinical studies involved OVX rats and measured anxiety with the elevated plus maze (EPM) or with the open field (OF) [64,65,66]. In the first study, a high-fat diet, regardless of the fat composition, had anxiolytic effects on Wistar rats [64], while in the second study the n-3 PUFA supplementation had anxiolytic effects in Sprague Dawley rats [65]. Finally, Da Rocha and colleagues [66] showed that the OVX group supplemented with both EPA and DHA 20 days before and 20 days after surgery did not differ from the control group in the EPM. In any case, they found that the supplemented OVX group moved significantly more in OF than the control OVX group, inferring that the supplementation with n-3 PUFA had an anxiolytic effect.

In the first study, the standard chow enriched with fish oil and administered for eight weeks after the ovariectomy stimulated the serotoninergic activity expressed by increased 5-hydroxyindoleacetic acid levels and serotonergic turnover [64].

In the second study, fish oil administered by gavage for 10 weeks after the ovariectomy maintained the normal homeostatic balance between pro-inflammatory and anti-inflammatory microglia phenotypes, and exerts antidepressant and neuroprotective activities, accompanied with neuroimmune-modulating actions [65].

The human study was a randomized placebo-controlled trial, where women aged 40–62 years daily received 1.8 g of n-3 PUFA (or placebo) for 12 weeks, and anxiety symptoms were evaluated by means of generalized anxiety disorder questionnaire (GAD-7). It was found that n-3 PUFA supplementation did not improve anxiety symptoms over placebo [70].

### 3.3. Effects of n-3 PUFA on Depression

Five studies on rodents and nine on humans which dealt with depression were carried out.

Most preclinical studies used female Wistar rats [64,66,67,68], except one study that used female Sprague Dawley rats [65]. Animals received n-3 PUFA via enriched diets for 12 weeks [67,68], 8 weeks [64] after the ovariectomy, via gavage for 10 weeks after the ovariectomy [65], or for 20 days before and after the ovariectomy [66]. All studies used different percentages and compositions of n-3 PUFA (Table 1).

These five preclinical studies evaluated the effects of diet on depressive behaviors by means of the Forced Swimming Test (FST) and one of these added the Sucrose Preference Test (SPT) [65]. While Dornellas and colleagues [64] showed no significant differences in FST parameters, four studies reported decreased immobility [65,66,67,68], and two of them described also increased climbing [67,68]. In particular, Choi and colleagues [68] reported that the supplementation with EPA or DHA, but not with ALA, decreased immobility and increased climbing. Wu and colleagues [65] corroborated the antidepressant effects, reporting the decreased latencies in the novelty-suppressed feeding test, but not in SPT. Interesting biochemical results reported that n-3 PUFA supplementation increased serum serotonin concentration and ER-α but not ER-β expression, and decreased brain levels of Prostaglandin Estradiol (PGE2). Furthermore, n-3 PUFA supplementation decreased n-6 PUFA levels in dose-dependent manner, increased cAMP response element binding protein (CREB) and BDNF hippocampal expression, and finally decreased tumor necrosis factor-α (TNF-α) and interleukin-6 (IL-6) hippocampal levels [67]. Daily supplementation with n-3 PUFA ameliorated the neuronal apoptosis and neuroimmune overactivation caused by ovariectomy [65].

As for human studies, literature screening produced three randomized controlled trials [70,71,75], one open-label trial [72], two cross-sectional studies [74,77], one cohort study [78] and two retrospective cohort studies [73,76].

Most studies considered the ethnicity of women and depressive symptoms (Table 4). In the three randomized controlled trials, n-3 PUFA supplementation consisted in: 350 mg EPA and 50 mg DHA three times a day for eight weeks [71]; capsules containing 425 mg of EPA, 100 mg DHA and 90 mg of other omega-3 three times a day (for a total amount of 1.8 g/day of n3-PUFA) for 12 weeks [70]; 20 mg of citalopram and 1 g of n-3 PUFA for one week [75]. In the open-label trial, n-3 PUFA supplementation consisted in capsules containing ethyl esters of EPA, approximately 465 mg per capsule, and DHA, approximately 375 mg per capsule (for a total amount of 2 g/day) twice a day for eight weeks [72]. While in Cohen and colleagues’ study [70] n-3 PUFA supplementation did not affect mood, in the study by Lucas et al. [71], n-3 PUFA supplementation ameliorated psychological distress and depressive symptoms in 55 women, aged 40–55 years. Moreover, a significant decrement in mean depression scores after two and four weeks of n-3 PUFA supplementation was described in 60 women, aged 45–65 years [75].

In the open-label trial [72], n-3 PUFA supplementation induced a significant decrease in depressive symptoms in 19 women aged 52.5 years on average.

In the Cross-Sectional studies, the n-3 PUFA dietary intake was assessed through phone interviews analyzed by using Can-pro 4.0 [74], 24-h recall method [78], or with a validated 147-item Food Frequency Questionnaires (FFQ) [77]. Nutritional values were assigned with DietSys software [77] and with the food composition table published by the Rural Development Administration [78]. In addition, Jin and colleagues [74] measured Red Blood Cells (RBC) fatty acid composition. Negative correlations between n-3 PUFA dietary intake and depressive symptoms were found in 3054 early perimenopausal women, aged 42–52 years [77]. Furthermore, a significant negative correlation between RBC levels of n-3 PUFA (ALA, DPA, and DHA) and depressive symptoms was found in women using HT [74]. In the Cohort study of Chae and Park [78], it was found a negative correlation between n-3 PUFA dietary intake and prevalence of depression in a dose-response manner in 4150 postmenopausal women aged 62–67 years.

Finally, in the two retrospective cohort studies, n-3 PUFA dietary intake was evaluated by means of Women’s Health Initiative (WHI) FFQ [73] and 120-item FFQ [76]. In particular, Colangelo and colleagues [76] assessed nutritional values (including n-3 PUFA) with Minnesota Nutrition Data System NDS software. Persons and colleagues [73] found a positive association between n-3 PUFA RBC levels (DHA, both EPA + DHA and total n-3 PUFA) and depressive symptoms, but this effect disappeared after data were adjusted for demographic and health behavior characteristics. Moreover, when FFQ scores were considered (total n-3 PUFA, DHA, and DHA + EPA dietary intake), a positive association between n-3 PUFA and depressive symptoms was found again. Similarly, Colangelo and colleagues [76] showed that 1080 HT non-user women, assuming a diet rich in DHA and EPA, had higher risk to develop depressive symptoms.

### 3.4. Effects of n-3 PUFA on Cognition

Regarding cognition, the literature screening produced 1 study on rodents and 2 on humans.

To evaluate memory, OVX Wistar rats (9–10-months of age) were fed with choline in combination with DHA and tested in the radial arm maze. The dietary supplementation ameliorated memory retention and enhanced the dendritic branching in hippocampal Cornu Ammonis (CA1 and CA3) pyramidal neurons, as well as BDNF levels, while no effect on serum E2 concentration was observed [69].

In human studies, the samples consisted of postmenopausal women, although none of these studies provided a definition of menopause or postmenopause (Table 3) [79,80]. Both studies [79,80] indicated where the subjects were recruited from but not their ethnicity (Table 4) and correlated the physiological levels of n-3 PUFA with the results of various cognitive tests (Table 5). In particular, Ammann and colleagues [79] in a retrospective cohort study did not report any significant correlation between blood levels of EPA and DHA and cognitive tests results while, the randomized, double-blind, controlled pilot study by Strike and colleagues [80] reported that 24-week supplementation with EPA and DHA, in combination with other nutraceuticals (which induced enhanced RBC levels of DHA) reduced the latency of psychomotor response on the motor screening task and improved verbal memory on the verbal recognition memory test.

## 4. Discussion

The present study was aimed at reviewing and evaluating the current scientific knowledge about the effects of n-3 PUFA on emotional dysregulation and cognition during menopausal transition. For this purpose, we carried out a search on two different databases. The publications on which this systematic review focused helped to shed light on the effects of n-3 PUFA in both humans and animal models.

### 4.1. n-3 PUFA and Anxiety

Preclinical studies investigating the n-3 PUFA effects on anxiety in OVX rats are fewer than studies on depression. In fact, the research found only three studies that address this variable [64,65,66], and in all three articles both anxiety and depression were evaluated. The three studies evaluated anxiety by means of EPM, and two of them agree with the anxiolytic effect of n-3 PUFA [64,65], while the study by Da Rocha and colleagues [66] reported the anxiolytic effect of n-3 PUFA only when anxiety was evaluated with the OF and not with the EPM. Likely, a reason for this discrepancy could be due to the differences in duration of n-3 PUFA supplementation. Indeed, in the two studies reporting an EPM anxiolytic effect, the supplementation occurred for at least eight weeks (8 weeks: [64]; 10 weeks: [65]) and treatment began in close proximity to ovariectomy [64,65], while in the study by Da Rocha and colleagues [66] the supplementation was shorter (40 days) and started 20 days before and lasted for 20 days after the ovariectomy.

Only one human study evaluated the effects of n-3 PUFA on anxiety (and depression) in menopausal women [70]. In contrast with the results of preclinical studies, this study reported that in a population of women in menopausal transition, the daily supplementation with n-3 PUFA did not improve anxiety symptoms. However, it must be noted that a very high percentage of the women encompassed in this study did not show any basal anxiety sign, suggesting that this basal condition does not allow to detect any n-3 PUFA effect on anxiety.

### 4.2. n-3 PUFA and Depression

Regarding the studies investigating n-3 PUFA effects on mood, most of the preclinical studies evidenced their beneficial effect on depressive symptoms [65,66,67,68], and only one did not show any effect of n-3 PUFA supplementation [64].

Conversely, human studies have produced mixed results. In fact, some studies showed beneficial effects of n-3 PUFA on depressive symptoms [71,72,74,75,77,78], while others surprisingly reported that n-3 PUFA may exert negative effects [73,76]. The only study reporting the absence of n-3 PUFA effect on depressive symptoms, mainly dealt with menopausal women without any substantial depressive symptom at baseline, not allowing the study to evidence any effect on depressive symptoms [70].

By analyzing n-3 PUFA effects on depressive symptoms specifically during menopause, we found that in three studies n-3 PUFA were administered through dietary supplementation [70,71,72], while in five study n-3 PUFA levels (detected through blood levels or inferred from diet questionnaires) were correlated with the results of questionnaires used to evaluate depression [73,74,76,77,78]. Beneficial effects on depressive symptoms were found in most of clinical trials, namely, in the studies in which n-3 PUFA were administered through dietary supplementation [71,72,75]. As for observational studies, two articles agree on negative correlation between n-3 PUFA levels and depressive symptoms assessed by questionnaires [77,78]. Notably, Jin and colleagues [74] found the same negative correlation only in the women using HT. And yet, other two observational studies highlighted the positive association between n-3 PUFA levels and depressive symptoms [73,76]. Therefore, some difference may be detected in the results of clinical trials and observational studies. In fact, clinical trials agree with the possibility of beneficial effects of n-3 PUFA [71,72,75], while observational studies report beneficial as well as negative effects [73,74,76,77,78].

However, in these studies, n-3 PUFA levels were evaluated through different versions of FFQ and none of these studies reported further measurements (such as blood levels) to confirm assessments. In conclusion, the different results obtained in menopausal women could be attributed not so much to the different effects of n-3 PUFA as to bias in sample selection or to poor control of confounding variables. As it is known, correlation does not imply causality, so further placebo-controlled clinical trials could be useful to resolve these contrasts. About this, the results of studies on the animal model offer greater reliability.

### 4.3. n-3 PUFA and Cognition

Regarding cognition, our research has found only one animal study [69]. In this case, n-3 PUFA dietary supplementation led to clear improvement in memory retention, accompanied by increased BDNF levels and dendritic branching in hippocampal CA1 and CA3 pyramidal neurons. However, it is necessary to consider the composition of the diet enriched with a combination of choline and DHA administered to rats not allowing to attribute the effects found to either specific element.

As for cognition investigated in humans, in the current research two studies were selected. In one of them [80] dietary supplementation with EPA and DHA improved verbal memory and decreased the psychomotor response. The other study failed to show any significant correlation between EPA and DHA levels and cognition [79].

### 4.4. n-3 PUFA and Biochemical Parameters

As regards the biochemical results of the preclinical studies, in spite of their heterogeneity numerous correlates have shown how much n-3 PUFA supplementation might affect brain biochemistry (Table 2). n-3 PUFA supplementation increased serum serotonin levels in two articles [67,68], and improved hippocampal serotoninergic activity and increased serotoninergic turnover in another study [64]. These results obtained in OVX rats are nicely supported by other studies which, although not investigating menopausal model, report the modulatory role of n-3 PUFA on serotoninergic system [55,81,82,83] and on depression [84,85,86]. In addition, n-3 PUFA enhance cell survival rate compromised by OVX. In fact, n-3 PUFA dietary supplementation reduced serum concentration of nitric oxide metabolites (NOx) [68] and number of apoptotic hippocampal cells, increased BDNF levels in hippocampus [67,68] and cerebral hemispheres [69], and increased hippocampal CREB [67,68] and pCREB [68].

Furthermore, n-3 PUFA supplementation increased hippocampal ER-α but not ER-β [67] and decreased brain tissue levels of PGE2 [67,68], while no effect was found on the serum E2 concentrations [69]. n-3 PUFA supplementation exerts its beneficial effects even regulating the neuroimmune system. In fact, reduction of pro-inflammatory markers whose increase was induced by OVX was reported [65,68]. Wu and colleagues [65] proved that n-3 PUFA supplementation increased the anti-inflammatory markers and restored the homeostatic functioning of microglia, whose alterations were induced by OVX.

Since menopause is related to marked changes in the immune system (increased pro-inflammatory markers) [87,88] the reduction of OVX-induced inflammation appears to support the therapeutic effects of n-3 PUFA in menopause.

## 5. Conclusions

This systematic review shows that the research on PubMed and Scopus yielded limited results compared to what would be expected, given the notoriety of the topics covered. However, it is important to consider that often during menopause may occur other important pathologies, such as cardiovascular problems, obesity, and bone problems, or even very severe diseases, such as cancer and neurodegenerative diseases, to which great concern is generally paid. Furthermore, in several of the analyzed studies menopause definition was missing or unclear, and this is a particularly relevant factor when comparing results in a systematic review. Therefore, the articles should be analyzed in a critical key, since in human studies samples composed of comparable populations would better highlight the results. Interestingly, preclinical studies may provide reliable information since they allow consistent control of variables (genetic background, sample representativity, results replicability). In fact, in the present research it was found superior coherence in the preclinical results in respect to the more divergent findings especially in the observational studies.

Taken as a whole, most preclinical and clinical studies highlight that, during menopause, the n-3 PUFA dietary supplementation beneficially affects anxiety, depression, and cognition, and exerts marked anti-inflammatory and cell survival-promoting effects. Although the described results are promising, the effects of controlled and regular intake of n-3 PUFA alone or in combination with other nutrients should be further analyzed to better understand its role on the psychological well-being of menopausal women.

## Figures and Tables

**Table 1:** Data extracted from rodent studies: topic, study design, age, sample and treatments.

Article	Main Topic	Study Design	Age-Strain	Ovariectomy	n-3 PUFA Treatment Details
Dornellas et al. [64]	Anxiety and Depression	Preclinical study	8 weeks old; Wistar Rats	X	Fish Diet: standard chow enriched with fish oil. The high-fat diets were prepared by adding, to the standard chow, 20% (*w*/*w*) fish oil, 20% (*w*/*w*) casein, 10% (*w*/*w*) sucrose, and 0.02% (*w*/*w*) butylated hydroxytoluene; duration: 8 weeks.
Wu et al. [65]	Anxiety and Depression	Preclinical study	12 weeks old; Sprague Dawley Rats	X	Refined fish oil administrated daily by gavage (1.5 g/kg, approximately 340 mg/g for EPA, 240 mg/g for DHA); duration: 10 weeks.
Da Rocha et al. [66]	Anxiety and Depression	Preclinical study	8 weeks old; Wistar Rats	X	Supplementation performed 20 days before and 20 days after the surgical procedure: daily dose of 500 mg/kg/day of omega-3 (1000 mg capsules, containing 180 mg of EPA and 120 mg of DHA); duration: 40 days.
Jin et al. [67]	Depression	Preclinical study	3 weeks old; Wistar Rats	X (after 8 weeks of supplementation)	The diets were isocaloric and modified with 0%, 1% or 2% EPA + DHA relative to the total energy intake (0 g, 8.09 g and 16.21 g of fish oil per kg of diet, respectively). The diets contained 10% of total energy from fat, with 42.94 g/kg diet of fish oil and grape seed oil; duration: 12 weeks.
Choi et al. [68]	Depression	Preclinical study	3 weeks old; Wistar Rats	X (after 8 weeks of supplementation)	Diets were isocaloric modified with 0% n-3 PUFA, 1% ALA, 1% EPA or 1% DHA relative to the total energy intake. The control diet contained 70 g/kg of grape seed oil; the ALA, EPA and DHA diets contained 8.27 g/kg of flaxseed oil, 4.46 g/kg of EPA EE and 4.67 g/kg of DHA EE, respectively. The control diet had 0.05% of n-3 PUFA, and the ALA, EPA and DHA diets had 6.40% of ALA, 6.35% of EPA and 6.35% of DHA in total fatty acids, respectively; duration: 12 weeks.
Konuri et al. [69]	Cognition	Preclinical study	9–10 months; Wistar Rats	X	Choline (4.6 mmol/kg/day) in combination with DHA (300 mg/kg/day); duration: 30 days.

Abbreviations: ALA, alpha lipoic acid; DHA, docosahexaenoic acid; EE, ethyl ester; EPA, eicosapentaenoic acid; n-3 PUFA, Omega-3 polyunsaturated fatty acids.

**Table 2:** Data extracted from rodent studies: analyses and main results of rodent studies.

Article	Behavioral Tests	Biochemical Analyses	Behavioral Results	Biochemical Results
Dornellas et al. [64]	EPM; FST Modified.	No Biochemical Analyses were performed.	High fat diet had an anxiolytic effect regardless the fatty acid composition. No differences were found in depressive-like behaviors.	In the hippocampus, fish oil diet induced a stimulation in the serotoninergic activity, which is expressed in an increase in 5-hydroxyindoleacetic acid levels and in serotonergic turnover.
Wu et al. [65]	EPM; FST; Sucrose Preference Test; Novelty Suppressed Feeding Test.	Hormone Assay: ELISA Kit for E2; Apoptosis analysis: terminal deoxynucleotidyl transferase-mediated FITC-dUTP nick end labelling (Tunel) method; Microglia activation: Immunostaining of Iba-1; Cytokine Expression and Microglia Polarization: Western blot analysis of phosphorylation of NF-κB pp65, p65, IκB, iNOS, Arg-1 and β-actin; RT-PCR analysis.	n-3 PUFA supplementation: reverted the OVX induced anxiety-like behaviors displaying notable anxiolytic properties; alleviated OVX induced depressive-like behaviors in the FST and NSFT.	n-3 PUFA supplementation increased: IL-10; IL-4; IκB; p65. n-3 PUFA supplementation decreased: IL-1β; IL-6; NFκB; n-3 PUFA supplementation ameliorated: microglia activation; neuronal apoptosis.
Da Rocha et al. [66]	EPM; FST; Open Field.	Thiobarbituric acid reactive substances and catalase in the brain tissue; Glutamate in the cerebrospinal fluid.	The n-3 PUFA supplementation had an anxiolytic effect increasing the locomotory activity in the OF. The depression-like behavior was improved in the FST. No differences between groups were found in the EPM.	n-3 PUFA supplementation did not had any effect on Thiobarbituric acid reactive substances, catalase and glutamate.
Jin et al. [67]	FST.	Gas chromatography for the fatty acid composition of the brain tissue; Brain tissue levels of PGE2; Immunofluorescence staining for ER-α and ER-β; Blood samples collection to measure: serotonin serum levels; plasma estrogen levels; Hippocampal Western blot analysis of: CREB; pCREB; TNF-α; BDNF; IL-1β, IL-6; ER-α or ER-β.	n-3 PUFA supplementation increased climbing and decreased immobility and had no significant effects on duration of swimming.	n-3 PUFA supplementation increased: serum serotonin concentrations; the brain phospholipid level of n-3 PUFA (20:5n3, 22:5n3 and 22:6n3) in a dose-dependent manner; expression of CREB (among 0% vs. 1% and 0% vs. 2%); expression of BDNF (among 0% vs. 2% and 1% vs. 2%); expression of ER-α (among 0% vs. 1% and 0% vs. 2%). n-3 PUFA supplementation decreased: PGE2 brain levels; brain phospholipid level of n-6 PUFA (20:4n6, 22:4n6 and 22:5n6) in a dose-dependent manner; TNF-α (among 0% vs. 2% and 1% vs. 2%); IL-6 (among 0% vs. 1% and 0% vs. 2%).
Choi et al. [68]	FST.	Gas chromatography for the fatty acid composition of the brain tissue; Plasma analysis for estrogens and malondialdehyde levels; Brain tissue levels of PGE2; Immunofluorescence staining for BDNF levels in DG. Serum analysis for: serotonin; NOx; superoxide dismutase levels. Hippocampal Western blot analysis for: CREB; pCREB; BDNF; TNF-α; IL-6; ER-α or ER-β. In vivo magnetic resonance imaging/spectroscopy of the left dorsal hippocampal region to calculate peak concentrations of: creatine; phosphocreatine; glucose; glutamate; *myo*-inositol.	Supplementation with EPA and DHA, but not ALA, decreased the duration of immobility by 49%, and increased climbing by 69%.	Supplementation with: ALA increased brain phospholipid proportion of 18:3n3 as compared to the control, EPA and DHA diet. ALA, EPA and DHA increased the brain phospholipid proportions of 20:5n3, 22:5n3 and 22:6n3, this increase was greater with EPA and DHA than ALA supplementation. ALA, EPA and DHA decreased the brain phospholipid proportions of 18:2n6, 20:4n6, 22:4n6 and 22:5n6 and the decrease of proportions of 20:4n6, 22:4n6 and 22:5n6 were greater with of EPA and DHA than with ALA. EPA and DHA, increase serum serotonin levels by 29%. EPA and DHA decreased: PGE2 brain levels by 37%; serum concentrations of NOx by 52%; TNF-α expression by 26%; IL-6 expression by 29%. EPA and DHA increased hippocampal expression: hippocampal expression of ER-α by 21%; CREB by 34%; pCREB by 56%; BDNF by 32%.
Konuri et al. [69]	Eight-arm radial maze test;	Right cerebral hemisphere BDNF analysis using ELISA kit. E2 serum levels measured with ELISA kit. Golgi-Cox staining of the left cerebral hemisphere to evaluate dendritic arborization and length.	The dietary supplementation of choline-DHA significantly improved the memory retention.	The dietary supplementation of choline-DHA: Increase BDNF levels; improved basal and apical dendritic branching points and dendritic intersections in CA1 and CA3; Did not show any effect on serum E2 concentration.

Abbreviations: Arg-1, Arginase-1; BDNF, brain-derived neurotrophic factor; CA, Cornu Ammonis; CREB, cAMP response element binding protein; DG, dentate gyrus; ELISA, enzyme-linked immunosorbent assay; EPM, elevated plus maze; ER, estrogen receptor; FST, forced swimming test; IL, interleukin; IκB, NF-κB inhibitor; NOx, nitrogen oxides; NSFT, novelty suppressed feeding test; OVX, ovariectomized; pCREB, phosphorylated CREB; PGE2, prostaglandin E2; pp65-p65 subunit; TNF, tumor necrosis factor.

**Table 3:** Schematic representation of definitions about menopause stages extracted from the 11 human studies which met the inclusion criteria of the present systematic review.

Article	MENOPAUSE Definitions
Cohen et al. [70]	Menopause transition: *amenorrhea ≥60 days in the past year* Postmenopause: *≥12 months since last menstrual period or bilateral oophorectomy* Hysterectomy: *with follicle stimulating hormone >20 mIU/mL and estradiol of ≤50 pg/mL*
Lucas et al. [71]	Postmenopausal status: *12 months of amenorrhea after the final menstrual period*
Freeman et al. [72]	Peri- Post- menopause: *Women that met perimenopause or postmenopause status as defined by the standardized Stages of Reproductive Aging Workshop criteria*
Persons et al. [73]	Postmenopause: not specified by authors.
Jin et al. [74]	Menopause: not specified by authors.
Masoumi et al. [75]	Postmenopause: *at least 12 months of amenorrhea.*
Colangelo et al. [76]	Postmenopause: *Women were classified as postmenopausal if (a) they responded ‘yes’ to the question, ‘Have you gone through menopause (change of life)?’, or (b) had a prior hysterectomy and bilateral oophorectomy.*
Li et al. [77]	Early Perimenopause: *menstrual bleeding in the past 3 months accompanied by changes in cycle regularity.* Premenopause: *menstrual bleeding in the past 3 months with no change in cycle regularity in the past 12 months.*
Chae and Park [78]	Menopause: not specified by authors. Postmenopause: not specified by authors.
Ammann et al. [79]	Postmenopause: not specified by authors.
Strike et al. [80]	Postmenopause: not specified by authors.

**Table 4:** Data extracted from human studies: topic, study design, age, sample and exclusion criteria.

Article	Main Topic	Study Design	Sample Size and Age (Years)	Ethnicity	Exclusion Criteria
Cohen et al. [70]	Anxiety and Depression	Randomized Controlled Trial	*n* = 355 Age: 40–62	White; African American; Other	Body Mass Index > 37; use of hormones or hormonal contraceptives in the past 2 months; use of prescription or over-the-counter treatments for vasomotor symptoms in the past month; any unstable medical conditions; contraindications to exercise training, yoga, or omega-3; current participation in regular exercise or yoga; current use of omega-3 supplements or frequent consumption of fish; MDE in the past three months.
Lucas et al. [71]	Depression	Randomized Controlled Trial	*n* = 120 Age: 40–55	White	Severe MDE [scores of 26 on HAM-D-21]; history of schizophrenia or bipolar I and II disorder; imminent risk of suicide or homicide; postmenopausal status for >5 years; medical conditions that affect mental health; substance abuse or dependence; fish allergies; high fish consumption (>3 servings/week) in the past 3 months; use of antidepressants; hormone replacement therapy; fish-oil supplements in the past 3 months; anticoagulants use.
Freeman et al. [72]	Depression	Open-Label Trial	*n* = 19 Age mean: 52.5 ± 4.9	Caucasian; African American; Other	Currently pregnant, breast-feeding, or trying to conceive; currently being treated with an antidepressant, hormone treatment, or n-3 PUFA supplements or with one of the preceding treatments within 1 month of study entry; suicidal ideation; current or recent (past month) diagnosis of panic disorder or obsessive-compulsive disorder or history of psychosis, mania, or hypomania, as assessed by the MINI, diagnosis of treatment-resistant Major Depressive Disorder; fish or fish oil allergies; responded to placebo [950% decrease in the MADRS].
Persons et al. [73]	Depression	Retrospective Cohort Study	*n* = 7086 Age: 63–81	Not specified	Not Specified.
Jin et al. [74]	Depression	Cross-Sectional Study	*n* = 214 Age: from 54.23 ± 5.43 to 56.02 ± 6.09	Koreans	Not Specified.
Masoumi et al. [75]	Depression	Randomized Controlled Trial	*n* = 60 Age: 45–65	Not specified	Depression scores higher than 30 at follow-ups and any known drug side effects.
Colangelo et al. [76]	Depression	Retrospective Cohort Study	*n* = 1616 Age: 45–84	Non-Hispanic White; African American; Chinese American; Hispanic	Not Specified.
Li et al. [77]	Depression	Cross-Sectional Study	*n* = 3054 Age: 42–52	Non-Hispanic White; Chinese; Japanese; Hispanic; Black	No intact uterus or ovaries; use of reproductive hormones and amenorrhea in the previous 3 months.
Chae and Park [78]	Depression	Cohort Study	*n* = 4150 Age: from 62.8 ± 0.3 to 67.1 ± 0.3	Korean	Men; pregnant, lactating, or premenopausal women; women with a total energy intake of less than 500 kcal or more than 5000 kcal/day; women with no data on depression.
Ammann et al. [79]	Cognition	Retrospective Cohort Study	*n* = 2157 Age: 65–80	USA	Not Specified.
Strike et al. [80]	Cognition	Randomized, Double-Blind, Placebo-Controlled Pilot Study	*n* = 27 Age: 60–84	English	Vestibular impairments; neurological disorder; lower limb surgery; allergy to seafood; regular consumption of multivitamin/fish oil supplements.

Abbreviations: HAM-D-21, 21-item Hamilton depression rating scale; MADRS, Montgomery-Asberg depression rating scale; MDE, major depressive episode; MINI, mini international neuropsychiatric interview.

**Table 5:** Data extracted from human studies: Analyses and main results of rodent studies.

Article	Main Topic	n-3 PUFA Treatment Details	Behavioral Analyses	Biochemical Analyses	Main Results
Cohen et al. [70]	Anxiety and Depression	1.8 g/day (3 pills/day, each containing 425 mg of EPA, 100 mg DHA and 90 mg of other omega-3) for 12 weeks.	Physician’s Health Questionnaire-8 (depression domains); Generalized Anxiety Disorder Questionnaire-7.	No Biochemical Analyses were performed.	n-3 PUFA did not improve mood over placebo.
Lucas et al. [71]	Depression	3 capsule/day containing 350 mg EPA and 50 mg DHA in the form of ethyl esters for 8 weeks.	MINI (version 5.0.0); Psychological General Well-Being Schedule; 20-item Hopkins Symptom Checklist Depression Scale; HAM-D-21; Clinical Global Impression Severity Scale; FFQ (based on marine products).	RBCs fatty acid composition.	Ethyl-EPA treatment over placebo improved significantly psychological distress and depressive symptoms in women without MDE.
Freeman et al. [72]	Depression	2 g/day (2 capsules per day each 1-g capsule contains 840 mg of the EE of n-3 PUFA, as a combination of EE of EPA (approximately 465 mg per capsule) and DHA (approximately 375 mg per capsule)) for 8 weeks.	MINI for the diagnosis of Major Depressive Disorder; MADRS.	RBCs fatty acid composition.	Significant decrease in MADRS scores after treatment.
Persons et al. [73]	Depression	No treatment has been used in this study.	Burnam 8-item scale for depressive disorders: combined CES-D/DIS short form.	RBCs fatty acid composition.	Positive association between: RBC n-3 PUFA levels (DHA, both EPA + DHA and total n-3 PUFA) and depressive symptoms (the effect disappeared after adjusting data for demographic and health behavior characteristics); n-3 PUFA dietary intake (total n-3 PUFA, DHA, and DHA + EPA) with a higher prevalence of depressive symptoms; the risk to develop depressive symptoms and total n-3 PUFA (in the follow-up analysis, after excluding prevalent cases of depression in baseline).
Jin et al. [74]	Depression	No treatment has been used in this study.	BDI; Medical Records to assess at least 3 HT use; Interviews to assess dietary intake and general information.	No Biochemical Analyses were performed.	Significant negative correlation between Erythrocyte levels of n-3 PUFA of ALA, DPA, and DHA and depression only in women using HT.
Masoumi et al. [75]	Depression	Citalopram with 1 g of n-3 PUFA for 1 week.	Diagnostic and Statistical Manual of mental disorders-IV questionnaire to assess depression; BDI.	No Biochemical Analyses were performed.	Mean depression score lower in two and four weeks after intervention.
Colangelo et al. [76]	Depression	No treatment has been used in this study.	FFQ modified; CES-D.	Blood collection for the assessment of E2.	Significant interaction of HT with n-3 PUFA intake and depressive symptoms.
Li et al. [77]	Depression	No treatment has been used in this study.	FFQ; CES-D.	No Biochemical Analyses were performed.	n-3 PUFA intake was negatively correlated with depressive symptoms in early perimenopausal but not in premenopausal women.
Chae and Park [78]	Depression	No treatment has been used in this study.	Self-reported mental health questionnaire to assess depression; 24-h phone call interview to assess dietary intake.	No Biochemical Analyses were performed.	n-3 PUFA intake in postmenopausal women was inversely proportional to depression in a dose-response manner.
Ammann et al. [79]	Cognition	No treatment has been used in this study.	Finger Tapping Test; Card Rotations Test; Benton Visual Retention Test; California Verbal Learning Test; Primary Mental Abilities (Vocabulary test); Letter and category fluency tests; Digit Span (Forward and Backward Test).	RBCs fatty acid composition.	RBC DHA-EPA levels were not significantly correlated with baseline cognitive function and cognitive change over time.
Strike et al. [80]	Cognition	4 capsules/day (1 g DHA and 160 mg EPA per day in addition to Ginkgo biloba, PS, α-tocopherol, folic acid, and vitamin B12) for 24 weeks.	Cambridge Cognition Ltd.: A battery of computer-based cognitive test; MOT; VRM; Paired Associate Learning; Stockings of Cambridge.	RBCs fatty acid composition.	Supplemented group had: shorter mean latencies in MOT; higher number of words remembered in the VRM.

Abbreviations: BDI, Beck depression inventory; CES-D/DIS, center for epidemiologic studies/diagnostic interview schedule; FFQ, food frequency questionnaire; HT, hormone therapy; MOT, motor screening task; RBCs, red blood cells; VRM, verbal recognition memory.

## Data Availability

Not applicable.
